# Emetic risk classification and evaluation of the emetogenicity of antineoplastic agents—updated MASCC/ESMO consensus recommendation

**DOI:** 10.1007/s00520-023-08220-5

**Published:** 2023-12-22

**Authors:** Karin Jordan, Alexandre Chan, Richard J. Gralla, Franziska Jahn, Bernardo Rapoport, Christina H. Ruhlmann, Paula Sayegh, Paul J. Hesketh

**Affiliations:** 1 Department of Hematology, Oncology and Palliative Medicine, Ernst von Bergmann Hospital Potsdam, Charlottenstraße 72, 14467 Potsdam, Germany; 2 Department of Medicine V, Hematology, Oncology and Rheumatology, University of Heidelberg, Heidelberg, Germany; 3 School of Pharmacy & Pharmaceutical Sciences, University of California Irvine, Irvine, CA USA; 4 Albert Einstein College of Medicine, Bronx, NY USA; 5 Department of Hematology and Oncology, Martin Luther University Halle-Wittenberg, Halle (Saale), Germany; 6 Medical Oncology Centre of Rosebank, Johannesburg, South Africa; 7 Department of Immunology, Faculty of Health Sciences, University of Pretoria, Pretoria, South Africa; 8 Department of Oncology, Odense University Hospital, Odense, Denmark; 9 Department of Clinical Research, University of Southern Denmark, Odense, Denmark; 10 OU Health Stephenson Cancer Center, Oklahoma City, OK USA; 11 Division of Hematology and Oncology, Lahey Hospital & Medical Center, Burlington, MA USA

**Keywords:** Emetogenicity, Nausea, Vomiting, Risk classification, Antineoplastic agents

## Abstract

**Purpose:**

Our goal was to identify new anticancer agents approved by the US Food and Drug Administration (FDA) and the European Medical Agency (EMA) since the 2016 MASCC/ESMO antiemetic update and classify their emetic potential.

**Methods:**

The MASCC/ESMO Expert Panel classified the emetogenicity of the identified new antineoplastic agents based on nonsystematic reviews of randomized controlled trials, analysis of product labeling, and evaluation of emetic classification in other international guidelines and informal consensus. The emetogenic classification system for oral anticancer agents was revised into two emetic risk categories (minimal–low; moderate–high) to be consistent with the system reported by ASCO (American Society of Clinical Oncology) in their 2017 guideline update. The previously employed four emetic risk classification categories for intravenously administered antineoplastic agents were retained for this update.

**Results:**

From June 2015 to January 2023, 107 new antineoplastic agents (44 intravenously administered and 63 orally administered agents) were identified. The reported incidence of vomiting varied significantly across studies for many agents, especially for oral anticancer agents.

**Conclusion:**

The MASCC/ESMO Expert Panel acknowledges the limitations of our efforts to classify the emetic potential of anticancer agents, especially the imprecision associated with oral agents. However, we have attempted to provide a reasonable approximation of the emetic risk associated with new antineoplastic agents by searching the available literature and reviewing other available international antiemetic guidelines.

## Introduction

In 1997, an emetogenic classification schema for anticancer agents was introduced and has formed the basis for subsequent antiemetic prophylaxis recommendations by guideline panels [4, 6]. Since the 2004 Perugia Antiemetic Consensus Conference, chemotherapy agents were divided into four categories based on the risk of emesis in the absence of antiemetic prophylaxis (Table 1) [9, 10]. Of note, nausea was not incorporated into this schema. Many new antineoplastic agents have been introduced since the last MASCC/ESMO antiemetic guideline update in 2016 [7, 11].

**Table 1:** MASCC/ESMO emetic risk groups 2016*

Intravenous agents	Emetic risk	Oral agents	Emetic risk
High	Risk in nearly all patients (> 90%)	High	Risk in nearly all patients (> 90%)
Moderate	Risk in 30 to 90% of patients	Moderate	Risk in 30 to 90% of patients
Low	Risk in 10 to 30% of patients	Low	Risk in 10 to 30% of patients
Minimal	Fewer than 10% at risk	Minimal	Fewer than 10% at risk

^*^Proportion of patients experiencing emesis in the absence of effective antiemetic prophylaxis. The incidence of nausea is not part of the risk classification

It remains a challenge to accurately define the emetic risk associated with antineoplastic agents [3, 8]. The data on emesis in various trials of anticancer agents are usually highly heterogenous (different tumor types, advanced versus non-advanced disease, systemic treatment naïve or previously treated, used alone or in combination with other agents, different antiemetic prophylaxis if given or not reported, different reporting system, e.g., CTCAE (Common Terminology Criteria of Adverse Events) all grades versus only grade 3/4). Oral anticancer agents provide additional challenges. Most oral agents tend to be used in extended regimens of daily use rather than the single bolus administration schedule commonly employed with intravenous agents. As these agents are typically administered continuously over protracted periods, traditional concepts of acute and delayed nausea and vomiting lose their relevance in these settings.

In the current update, the following questions related to antineoplastic agent emetogenicity were addressed:

1. Identify new antineoplastic agents approved by the FDA and/or EMA since the last update (time frame: June 2015 to January 2023).
2. Characterize the emetic potential of new intravenously administered antineoplastic agents and place them at an appropriate level in the four-level classification schema.
3. Modify the original four-level classification system for oral agents to a two-level system (minimal to low and moderate to high) [5] and place both prior and new oral antineoplastic agents into the appropriate level.

## Methods

As the initial step, new antineoplastic agents approved by the FDA and/or EMA since the last update from June 2015 to January 2023 (data cut off) were identified by two independent reviewers. The data source was the FDA summary (https://www.fda.gov/drugs/development-approval-process-drugs/new-drugs-fda-cders-new-molecular-entities-and-new-therapeutic-biological-products) and the EMA summary (https://www.ema.europa.eu/en/medicines/field_ema_web_categories%253Aname_field/Human/search_api_aggregation_ema_therapeutic_area_name/Cancer/field_ema_public_date/%5B2022-05-31T22%3A00%3A00Z%20TO%202023-01-20T22%3A59%3A59Z%5D?sort=field_ema_computed_date_field&order=desc).

Next, information on the incidence of vomiting was obtained by (1) a nonsystematic review of randomized controlled trials, (2) a review of information available in the summary of product characteristics, and (3) through informal consensus of the panel members. In addition, a detailed comparison of emesis classification schemas in the updated ASCO and NCCN antiemetic guidelines was conducted. In cases where data was inconclusive, the corresponding pivotal key phase II/III studies of the respective antineoplastic agent were reviewed. If clinical studies of an antineoplastic agent showed broad differences in the incidence of vomiting, results of the “worst outcome” were selected.

The intravenous anticancer agents were classified as being at minimal, low, moderate, or high emetic risk in accordance with the summarized vomiting rates.

Oral anticancer agents were placed into one of two emetic categories, minimal–low risk and moderate–high risk (Table 2). Of note, the emetic risk classification only refers to adult patients.

**Table 2:** MASCC/ESMO emetic risk groups 2023*

Intravenous agents	Emetic risk	Oral agents**	Emetic risk
High	Risk in nearly all patients (> 90%)	High/moderate	Risk in more than 30% of patients
Moderate	Risk in 30 to 90% of patients		
Low	Risk in 10 to 30% of patients	Low/minimal	Risk in fewer than 30% of patients
Minimal	Fewer than 10% at risk		

^*^Proportion of patients experiencing emesis in the absence of effective antiemetic prophylaxis. The incidence of nausea is not part of the risk classification

^**^The emetic potential of the oral anticancer agents is based upon a full course of therapy and not a single dose within the first cycle

## Results

Within the defined time frame, 107 new antineoplastic agents were identified. The reported incidence of vomiting varied considerably across studies, especially for oral anticancer agents. The emetic potential of the oral anticancer agents was based upon a full course of therapy and not a single dose.

All agents in Tables 3 and 4 are listed in alphabetical order.

**Table 3:** Emetogenic potential of single intravenous antineoplastic agents

High	Anthracycline/cyclophosphamide combination^a^ Carmustine Chlormethine (mechlorethamine) Cisplatin Cyclophosphamide ≥ 1500 mg/m^2^ Dacarbazine Streptozocin	
Moderate	Alemtuzumab Arsenic trioxide Azacitidine Bendamustine Busulfan Carboplatin* Clofarabine Cyclophosphamide < 1500 mg/m^2^ Cytarabine > 1000 mg/m^2^ Cytarabine/daunorubicin liposomal Daunorubicin Dinutuximab beta Doxorubicin Epirubicin	Idarubicin Ifosfamide Irinotecan Irinotecan peg-liposomal Lurbinectedin Naxitamab Oxaliplatin Romidepsin Sacituzumab-govitecan** Temozolomide^b^ Thiotepa^c^ Trabectedin Trastuzumab-deruxtecan**
Low	Aflibercept Amivantamab Axicabtagene-ciloleucel Belinostat Blinatumomab Bortezomib Brentuximab-vedotin Cabazitaxel Carfilzomib Catumaxomab Cetuximab Copanlisib Cytarabine ≤ 1000 mg/m^2^ Decitabine Docetaxel Doxorubicin peg-liposomal Elotuzumab Enfortumab-vedotin Eribulin Etoposide 5-Fluorouracil Gemcitabine Gemtuzumab-ozogamicin Inotuzumab-ozogamicin Isatuximab	Ixabepilone Loncastuximab-tesirine Margetuximab Melphalan-flufenamide Methotrexate Mirvetuximab-soravtansine Mitomycin Mitoxantrone Moxetumomab-pasudotox Necitumumab Nelarabine Paclitaxel Paclitaxel nab-albumin Panitumumab Pemetrexed Pertuzumab Tafasitamab Tagraxofusp Teclistamab Temsirolimus Tisagenlecleucel Tisotumab-vedotin Topotecan Trastuzumab-emtansine Vinflunine
Minimal	Asparaginase^#^ Atezolizumab Avelumab Belantamab-mafodotin Bevacizumab Bleomycin Cemiplimab Cladribine (2-chlorodeoxyadenosine) Daratumumab Dostarlimab Durvalumab Emapalumab Fludarabine Ipilimumab Mosunetuzumab	Nivolumab Obinutuzumab Ofatumumab Pembrolizumab Pixantrone Polatuzumab-vedotin Pralatrexate Ramucirumab Rituximab Trastuzumab Tremelimumab Vinblastine Vincristine Vinorelbine

^a^The combination of an anthracycline and cyclophosphamide in patients with breast cancer is highly emetogenic

^b^No direct evidence found for temozolomide IV; as all sources indicate a similar safety profile of oral temozolamide, the classification was based on oral temozolomide

^c^Classification refers to individual evidence from pediatric trials

^*^Emetic potential appears to be at the high end of the moderate category

^**^Emetic potential appears to be at the high end of the moderate category, most closely resembling that of carboplatin

^#^Asparaginase erwinia chrysanthemi (crisantaspase) and asparaginase (calaspargase pegol)

**Table 4:** Emetogenic potential of single oral antineoplastic agents*

High/moderate	Abemaciclib Adagrasib Avapritinib Bosutinib Cabozantinib Ceritinib Crizotinib Cyclophosphamide Enasidenib Fedratinib Hexamethylmelamine Imatinib	Lenvatinib Lomustine Midostaurin Mobocertinib Niraparib Olaparib Procarbazine Ribociclib Rucaparib Selinexor** Temozolomide Vinorelbine
Low/minimal	Acalabrutinib Afatinib Alectinib Alpelisib Apalutamide Asciminib Axitinib Bexarotene Brigatinib Capecitabine Capmatinib Chlorambucil Cobimetinib Dabrafenib Dacomitinib Darolutamide Dasatinib Duvelisib Encorafenib Entrectinib Erdafitinib Erlotinib Estramustine Etoposide Everolimus Fludarabine Futibatinib Gefitinib Gilteritinib Glasdegib Hydroxyurea Ibrutinib Idelalisib Infigratinib Ivosidenib Ixazomib Lapatinib Larotrectinib Lenalidomide Lorlatinib Melphalan (L-Phenylalanine mustard)	Methotrexate Neratinib Nilotinib Nintedanib Olutasidenib Osimertinib Palbociclib Panobinostat Pazopanib Pemigatinib Pexidartinib Pomalidomide Ponatinib Pralsetinib Regorafenib Relugolix Ripretinib Ruxolitinib Selpercatinib Sonidegib Sorafenib Sotorasib Sunitinib Talazoparib Tazemetostat Tegafur/uracil Tepotinib Thalidomide Tioguanin (6-thioguanine) Tivozanib Topotecan Trametinib Trifluridine/tipiracil Tucatinib Umbralisib Vandetanib Vemurafenib Venetoclax Vismodegib Vorinostat Zanubrutinib

^*^Classified emetic potential of oral agents based upon a full course of therapy and not a single dose within the first cycle

^**^Emetic potential appears to be at the high end of the moderate category

### For intravenous agents

No highly emetogenic intravenous agents were identified. Eight moderately emetogenic intravenous agents were identified (arsenic trioxide, cytarabine/daunorubicin liposomal, dinutuximab beta, irinotecan peg-liposomal, lurbinectedin, naxitamab, sacituzumab-govitecan, trastuzumab-deruxtecan). For sacituzumab-govitecan and trastuzumab-deruxtecan, the emetic potential appears to be at the high end of the moderate category, most closely resembling that of carboplatin. As such, those two new classified agents received an asterisk in the table to highlight this point.

Twenty-one intravenous agents were classified as low emetogenic (amivantamab, axicabtagene-ciloleucel, copanlisib, decitabine, elotuzumab, enfortumab-vedotin, gemtuzumab-ozogamicin, inotuzumab-ozogamicin, isatuximab, loncastuximab-tesirine, margetuximab, melphalan-flufenamide, mirvetuximab-soravtansine, moxetumomab-pasudotox, necitumumab, nelarabine, tafasitamab, tagraxofusp, teclistamab, tisagenlecleucel, tisotumab-vedotin). Fifteen intravenous agents were classified as minimally emetogenic (asparaginase,^1^ atezolizumab, avelumab, belantamab-mafodotin, cemiplimab, daratumumab, dostarlimab, durvalumab, emapalumab, ipilimumab, mosunetuzumab, obinutuzumab, polatuzumab-vedotin, ramucirumab, tremelimumab).

### For oral agents

Fourteen oral agents were identified as high–moderate (abemaciclib, adagrasib, avapritinib, cabozantinib, enasidenib, fedratinib, lenvatinib, lomustine, midostaurin, mobocertinib, niraparib, ribociclib, rucaparib, selinexor). Selinexor also received an asterisk to indicate the emetic potential to be at the higher end of the high–moderate risk category. Forty-nine agents were classified as low–minimal (acalabrutinib, alectinib, alpelisib, apalutamide, asciminib, bexarotene, brigatinib, capmatinib, cobimetinib, dacomitinib, darolutamide, duvelisib, encorafenib, entrectinib, erdafitinib, estramustine, futibatinib, gilteritinib, glasdegib, infigratinib, ivosidenib, ixazomib, larotrectinib, lorlatinib, neratinib, nintedanib, olutasidenib, osimertinib, palbociclib, panobinostat, pemigatinib, pexidartinib, pralsetinib, relugolix, ripretinib, selpercatinib, sonidegib, sotorasib, talazoparib, tazemetostat, tepotinib, tivozanib, topotecan, trametinib, trifluridine/tipiracil, tucatinib, umbralisib, venetoclax, zanubrutinib).

### Combination antineoplastic regimens

For combination antineoplastic regimens, the emetic level is determined by identifying the most emetic agent in the combination. One exception to this rule remains the combination of cyclophosphamide and anthracycline (AC regimen). Both are moderately emetogenic agents; however, the regimen is highly emetic when coadministered. It has to be acknowledged that the studies defining the AC regimens as highly emetogenic were conducted almost exclusively in women with breast cancer. It is still a matter of debate whether AC used as a component of combination regimens such as the CHOP (doxorubicin plus cyclophosphamide, vincristine, and prednisone) regimen in patients with non-Hodgkin lymphomas is also highly emetic.

## Discussion

Classifying antineoplastic agents according to their emetic potential remains imprecise and challenging. This process is hindered by the fact that the potential of an administered antineoplastic agent to cause emesis has been established rigorously for only a few agents. Due to limitations further discussed below, the third Antiemetic Perugia Consensus Conference decided to change from the original Hesketh classification schema from 1997 with five emetic risk groups to four broad emetogenic risk groups (high, moderate, low, minimal) [2, 6].

During the classification process, the following challenges noted during prior guideline updates were continuously present:

- A lack of specific information on nausea/vomiting in clinical trial publications,
- Listing only CTCAE grade 3/4 nausea and/or vomiting or the combination of both,
- Reporting all grades only of nausea and/or vomiting,
- Not specifying the observation period when the toxicity data were collected,
- Missing information on whether antiemetic prophylaxis or treatment was administered,
- Limited data for single antineoplastic agents as many agents are given as combination regimens,
- Inclusion of heavily pre-treated patient populations makes it difficult to differentiate whether the vomiting is due to the antineoplastic agent or due to advanced cancer itself (example: imatinib in CML, chronic phase imatinib is of low emetogenic potential, in blast crisis, imatinib is of moderate emetogenic potential),
- Lack of information about intercurrent illnesses or concomitant medications, which cause nausea and emesis,
- Failure to report the time frame of emetic outcomes, thus providing little basis to determine the potential of a new antineoplastic agent to induce acute or delayed nausea and vomiting or even anticipatory nausea and vomiting,
- No detailed information about patient-related variables in correlation to the incidence of nausea and vomiting, such as sex, age, anxiety, and history of alcohol consumption,
- The tendency to underestimate the incidence of emesis that occurs in the days after the patient has left the clinic and is no longer under direct observation.

The reported incidence of vomiting with three new antineoplastic agents (sacituzumab-govitecan, trastuzumab-deruxtecan, and selinexor) deserves special mention. The two intravenously administered agents (sacituzumab-govitecan and trastuzumab-deruxtecan) warrant classification in the high–moderate emetogenic range analogous to carboplatin. The oral agent selinexor warrants classification in the higher end of the moderate–high-risk category.

Characterizing emetic potential for oral antineoplastic agents is especially problematic and challenging. These agents are typically administered chronically over protracted periods. Traditional concepts of acute and delayed nausea and vomiting lose their relevance in these settings.

One other limiting factor is the standard toxicity reporting systems. In clinical studies, the CTCAE criteria are often used (Table 5). For example, CTCAE grade 1 describes 1–2 episodes of vomiting in 24 h. Although this information would be critically important in evaluating the emetic potential of a given agent, grade 1 and 2 CTCAE toxicities are rarely reported in publications. The suggestion of the prior MASCC/ESMO guideline panel to record the frequency and intensity of nausea and vomiting using standard antiemetic methodology rather than the less informative Common Terminology Criteria was never adopted in clinical trials [2]. Further, the CTCAE criteria represent a classical clinician-reported outcome, and it is well-known that clinicians often underreport symptoms experienced by the patient [1]. In contrast, patient-reported outcomes usually identify a higher incidence and severity of treatment-related symptoms.

**Table 5:** Common terminology criteria: term vomiting

Adverse event	Grade				
	1	2	3	4	5
Vomiting*	1 – 2 episodes (separated by 5 min) in 24 h	3 – 5 episodes (separated by 5 min) in 24 h	≥ 6 episodes (separated by 5 min) in 24 h; tube feeding, TPN or hospitalization indicated	Life-threatening consequences; urgent intervention indicated	Death

^*^Definition: A disorder characterized by the reflexive act of ejecting the contents of the stomach through the mouth

Finally, it should be acknowledged that the MASCC/ESMO antiemetic prophylaxis guideline recommendations at present can only be applied to intravenously administered antineoplastic agents, given the paucity of antiemetic trials specifically designed for orally administered antineoplastic agents.

Of note, several intravenous agents (carboplatin, sacituzumab-govitecan, trastuzumab-deruxtecan) are significantly more emetogenic than most moderate agents and may warrant consideration to be classified in a separate category between the moderate and high categories in the future. This may allow more precise antiemetic prophylaxis recommendations.

Acknowledging these limitations, we have attempted to provide a reasonable approximation of the emetic risk associated with systemic antineoplastic agents. Ultimately, this process will only improve if appropriate information on nausea, vomiting, and concomitant medication (e.g., antiemetic prophylaxis or treatment) is collected and reported for phase II and III clinical studies in new antineoplastic agents.

## Footnotes

1 asparaginase erwinia chrysanthemi (crisantaspase) and asparaginase (calaspargase pegol)
